# Fine-tuning the lysine-arginine duet: a dietary approach to suppress tumor growth

**DOI:** 10.1186/s43556-026-00588-0

**Published:** 2026-09-22

**Authors:** Ben-Chi Zhao, Xin-Yuan Zhao, Wen-Xiu Li, Xiao Lan, Xiao-Ming Huang, Hao Feng, An-Qi Li, Run-Lu Shi, Hui-Lin Li, Xi Xie, Guo-Hui Wan, Ren-Jie Jiao, Di Chen, Jian-Sheng Kang, Qiao-Ping Wang

**Affiliations:** 1 Laboratory of Metabolism and Aging, School of Pharmaceutical Sciences, Shenzhen Campus of Sun Yat-Sen University, Shenzhen, 518107 China; 2 Clinical Systems Biology Laboratories, The First Affiliated Hospital of Zhengzhou University, Zhengzhou, 450052 China; 3 Department of Neurology, The First Affiliated Hospital of Zhengzhou University, Zhengzhou, 450052 China; 4 The First Clinical College, Zhengzhou University, Zhengzhou, 450052 China; 5 Sino-French Hoffmann Institute, School of Basic Medical Science, Guangzhou Medical University, Guangzhou, 511436 China; 6 State Key Laboratory of Metabolic Dysregulation & Prevention and Treatment of Esophageal Cancer, The First Affiliated Hospital of Zhengzhou University, Zhengzhou, 450052 China; 7 State Key Laboratory of Optoelectronic Materials and Technologies, Guangdong Province Key Laboratory of Display Material and Technology, School of Electronics and Information Technology, Sun Yat-Sen University, Guangzhou, 510006 China; 8 School of Pharmaceutical Sciences, Sun Yat-Sen University, Guangzhou, 510006 China; 9 Guangdong Provincial Key Laboratory of Diabetology, Guangzhou Key Laboratory of Mechanistic and Translational Obesity Research, The Third Affiliated Hospital of Sun Yat-Sen University, Guangzhou, 510630 China; 10 State Key Laboratory of Anti-Infective Drug Discovery and Development, School of Pharmaceutical Sciences, Sun Yat-Sen University, Guangzhou, 510006 China

**Keywords:** Lysine, Arginine, Dietary approach, Tumor, Cancer-immune crosstalk, Immune-nutritional crosstalk

## Abstract

While dietary therapies targeting individual amino acids in cancer show promise in modulating tumor metabolism, the issue of nutrient competition between tumors and the immune system remains unresolved. The current approaches face significant challenges, particularly in the context of metabolic adaptability and immune responses. Here, we demonstrate that the combined modulation of lysine (Lys) and arginine (Arg) balances tumor suppression and host fitness. Using a cross-species approach combining *Drosophila* and mouse models, we define a narrow therapeutic window for Lys and Arg. A balanced combination (0.5% Lys/0.5% Arg) reduces tumor burden, extends survival, and preserves host fitness in tumor-bearing mice, whereas an imbalanced formulation (0.25% Lys/1% Arg, LLHA) paradoxically accelerates tumor progression. Single-cell RNA sequencing revealed that LLHA was associated with increased B cells, M1-annotated macrophages and reduced T cells, consistent with systemic immune remodeling that may favor tumor progression, accompanied by decreased *Rps6* and *Rps29* and increased *Hba-a1* and *Hbb-bt* expression, suggesting alterations in mechanistic target of rapamycin (mTOR), hemoglobin, and ribosomal/translational pathways in immune cells. By defining biphasic thresholds and identifying optimal Lys/Arg combinations, this work provides a preclinical framework for investigating how balanced Lys/Arg modulation may influence tumor control and host fitness. This represents a step towards potential future synergy with immunotherapies, contingent upon further validation of immune dependency.

**Supplementary Information:**

The online version contains supplementary material available at https://doi.org/10.1186/s43556-026-00588-0.

## Introduction

The relentless progression of cancer underscores the urgent need for therapies that go beyond traditional interventions [1–3]. Amino-acid metabolism underpins cancer metabolic reprogramming by fueling protein and nucleotide synthesis, redox balance, epigenetic regulation, and amino-acid sensing, while simultaneously governing immune-cell activation and effector function, thereby bridging tumor-intrinsic metabolism with tumor–host immune competition [4–6]. Dietary optimization, particularly the targeted adjustment of key amino acids, has emerged as a promising strategy to slow tumor growth [4, 7–12].

Among these, Lys [13, 14] and Arg [15–17], two amino acids central to metabolism and immunity, have garnered attention for their potential role in cancer treatment [18]. Lys, an essential amino acid, exhibits tumor-suppressive potential under dietary restriction [19–21], but risks undernutrition and weakened immunity. In contrast, Arg, a conditionally essential amino acid, not only fuels tumor proliferation but also enhances immune response [22, 23]. Arg availability can therefore have context-dependent effects, potentially supporting tumor growth while also contributing to immune-cell function [24]. These contrasting properties suggest that the biological consequences of manipulating Lys or Arg may depend on both dose and host context.

Despite increasing interest in individual Lys restriction and Arg supplementation, the consequences of their combined modulation remain poorly understood. Lys and Arg can share cationic amino-acid transport systems, including SLC7A1 [25], providing a potential basis for interaction in nutrient availability and cellular responses. However, previous studies have largely examined these amino acids separately, leaving unresolved whether specific Lys/Arg combinations can suppress tumor growth while preserving host fitness. This question is particularly relevant because Lys and Arg differ in essentiality, endogenous availability, and immunometabolic functions, such that changing one amino acid may alter the biological effect of the other. We therefore reasoned that systematic mapping of combined Lys/Arg conditions could reveal response patterns that would not be apparent from single-amino-acid interventions alone.

To determine how combined Lys/Arg modulation influences tumor and host outcomes, we employed a cross-species response-mapping strategy consisting of a 36-combination Lys/Arg screen in a Drosophila RasV12-GFP tumor model followed by a 3 × 3 Lys/Arg supplementation matrix in B16F10 melanoma-bearing mice. Tumor volume, body weight, and survival were evaluated in the mouse model, and splenic single-cell RNA sequencing was used to characterize immune changes associated with contrasting tumor responses. The combined analysis identified 0.5% Lys/0.5% Arg as the most favorable overall regimen for survival and body-weight maintenance, whereas 0% Lys/1% Arg produced the strongest tumor suppression and 0.25% Lys/1% Arg showed the weakest tumor inhibition. Splenic scRNA-seq further revealed Lys/Arg-associated changes in B-cell, T-cell, and macrophage populations. Together, these findings provide a preclinical response-mapping framework for investigating how combined Lys/Arg modulation influences tumor and host phenotypes and for guiding future validation of amino-acid modulation strategies in cancer.

## Results

### Optimal combination of Lys and Arg enhances survival in tumor-bearing *Drosophila*

**Fig. 1:**
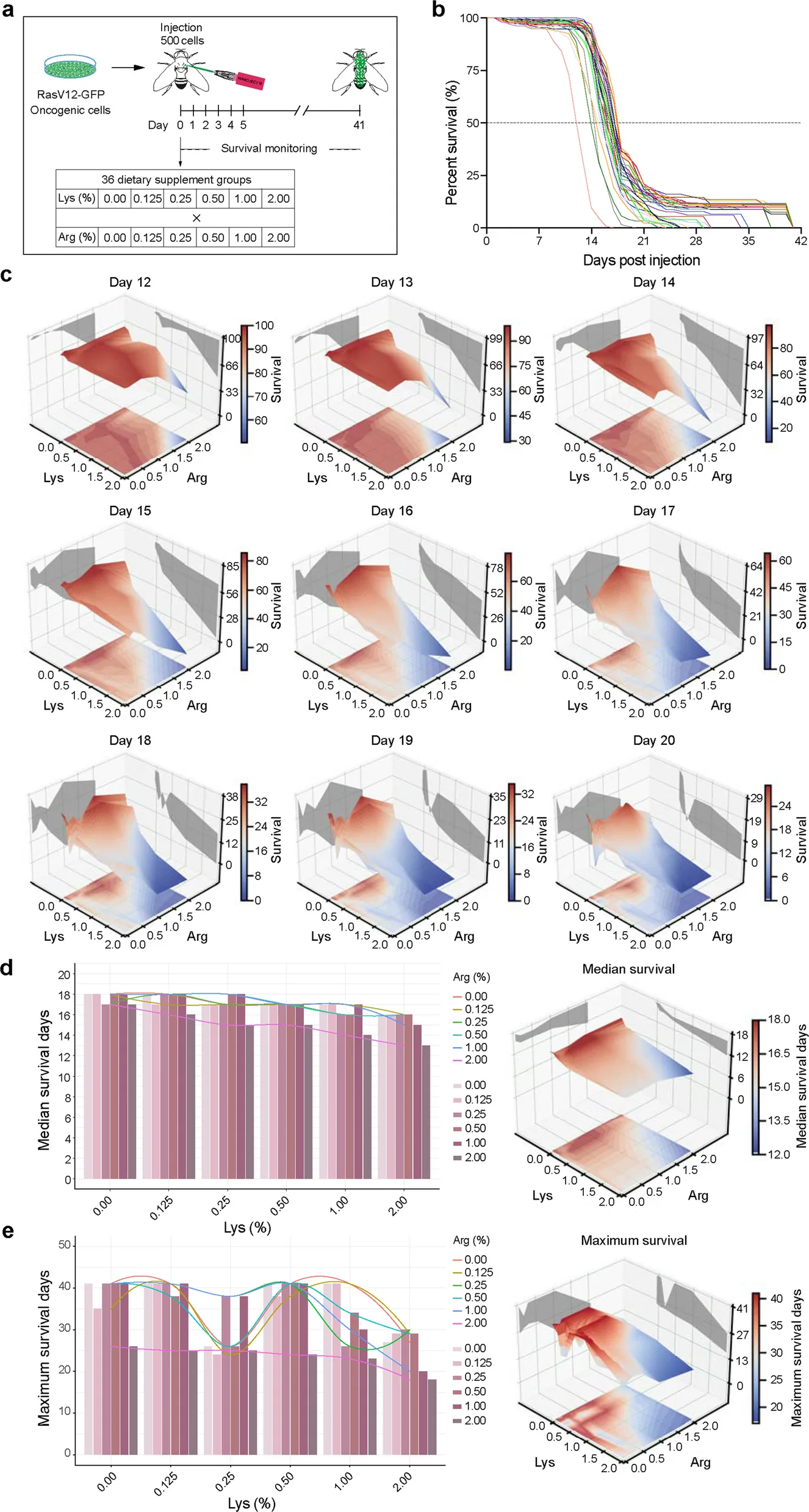
Optimal combination of Lys and Arg enhances survival in tumor-bearing *Drosophila.* **a** Schematic diagram of the *Drosophila* dietary screening design. A total of 500 RasV12-GFP oncogenic cells were injected into 3–5-day-old adult flies to establish the tumor-bearing fly model. After injection, tumor-bearing flies were assigned to 36 distinct Lys/Arg dietary regimens for 20 days. The 36 diets were generated from six Lys concentrations and six Arg concentrations, ranging from 0 to 2%, in a yeast-supplemented basal diet. **b** Survival curves of tumor-bearing flies receiving the 36 Lys/Arg dietary regimens. Survival was monitored daily after RasV12-GFP cell injection (*n* = 120). **c** 3D surface response analysis of percent survival from day 12 to day 20. The x- and y-axes indicate dietary Lys and Arg concentrations, respectively, and the z-axis represents percent survival. This analysis was used to visualize the dose-dependent interaction between Lys and Arg on survival. **d** Median survival analysis. The left panel shows median survival, defined as the day at which 50% mortality occurred, under each Lys/Arg combination. The right panel shows the corresponding 3D surface plot of median survival across the Lys/Arg matrix. **e** Maximum survival analysis. The left panel shows maximum survival under each Lys/Arg combination. The right panel shows the corresponding 3D surface plot of maximum survival across the Lys/Arg matrix. The schematic in panel (**a**) was created by the authors using Adobe Illustrator. Lys, lysine; Arg, arginine

To investigate the interplay between Lys and Arg in tumor suppression, we utilized a *Drosophila* model that allows for precise dietary manipulation. Thirty-six distinct diets were formulated with varying Lys and Arg concentrations (0.0%−2.0%), within a yeast-supplemented basal diet (0.023% Lys and 0.017% Arg). This design ensured adequate nutrient provision while isolating the antitumor effects of Lys and Arg (Fig. S1). Tumor progression was assessed by inoculating flies with oncogenic RasV12-GFP cells and assigning them to dietary regimens (Fig. 1a). The balance of dietary Lys and Arg significantly influenced survival in tumor-bearing *Drosophila* (Fig. 1b). Surface response analysis revealed a nonlinear interaction between these amino acids (Fig. 1c). Lys alone relatively exhibited a linear dose–response relationship, with optimal survival observed at low Lys concentrations (0.0–0.5%) with an exception at 0.25%, which reduced survival at a later stage. In contrast, Arg alone displayed a bell-shaped dose–response curve: peaking at 1% for maximal survival. Strikingly, high Lys (1.0–2.0%) combined with high Arg (1.0–2.0%) markedly reduced survival, with the poorest outcomes observed at the combination of 2% Lys and 2% Arg. Conversely, combining 0.0–0.5% Lys with 1% Arg maximized survival, indicating a favorable combination-dependent effect (Fig. 1c). These findings were corroborated by median survival analysis (day of 50% mortality) (Fig. 1d) and maximum survival (Fig. 1e), further emphasizing the critical role of Lys-Arg balance for survival. Together, the survival curve, median survival, maximum survival, and 3D response surface analyses indicate that Lys and Arg interact nonlinearly to shape survival outcomes in tumor-bearing *Drosophila*. The survival benefit was not driven by increasing either amino acid alone, but depended on a balanced Lys/Arg combination, with low Lys and moderate Arg producing the most favorable survival profile. Collectively, our results identify a dietary regimen of Lys (0.0%−0.5%, except 0.25%) combined with Arg (1.0%) as optimal for enhancing survival in tumor-bearing *Drosophila*, underscoring the importance of amino acid balance in dietary strategies for cancer intervention.

### Balancing Lys and Arg optimize survival in tumor-bearing mice

**Fig. 2:**
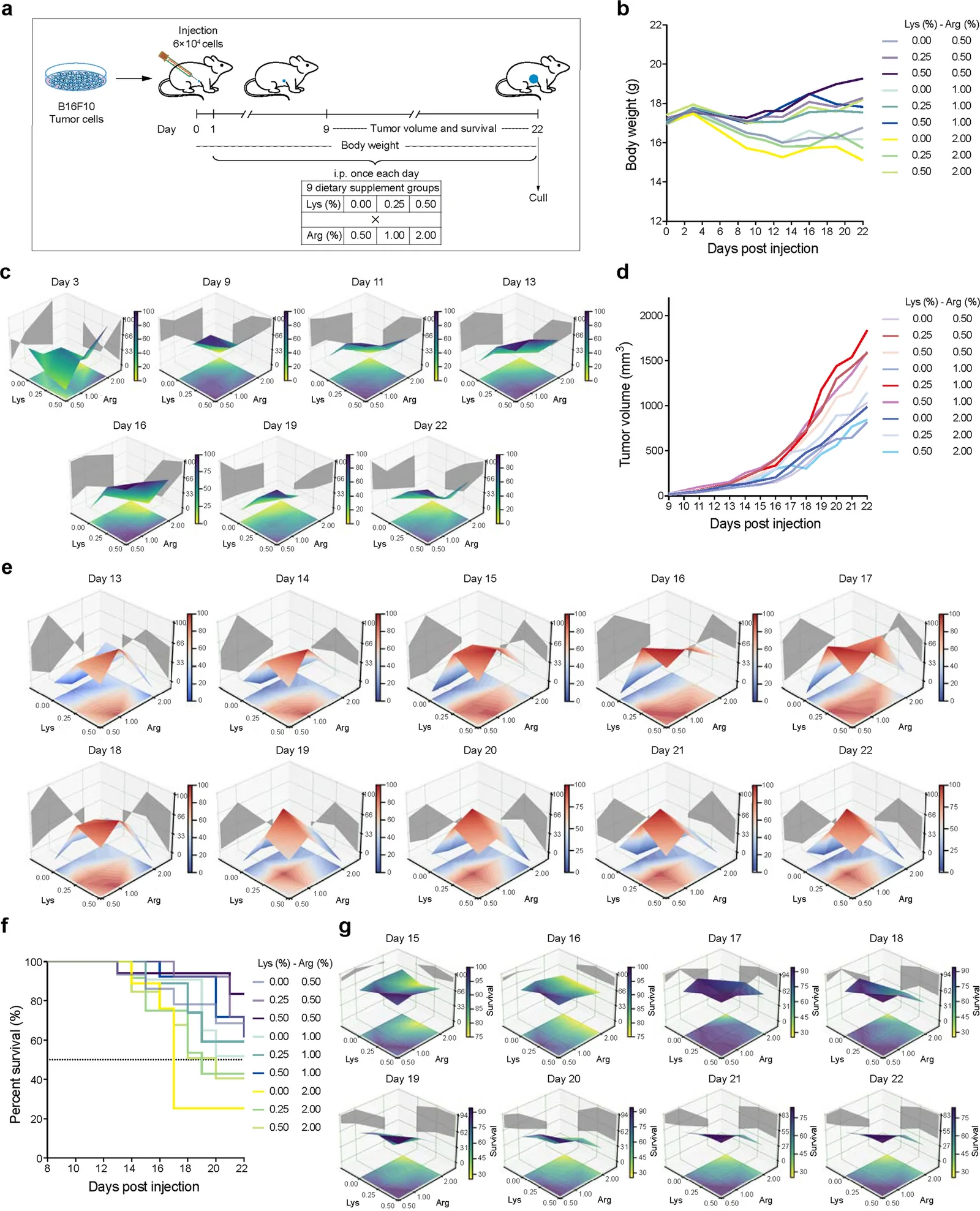
Balancing Lys and Arg optimize survival in tumor-bearing mice. **a** Schematic diagram of the experimental design. B16F10 cells were subcutaneously injected into an 8-week-old female C57BL/6 J mouse to establish a cell-line-derived xenograft (CDX) model, then the tumor-bearing mice were fed Lys-/Arg- free diets with i.p. 9 Lys-Arg dietary supplement regimens once each day from day1-22. The body weights of mice were assessed daily from day 0 to day 22. Tumor dimensions were assessed daily from day 9 to day 22 post-inoculation. **b** Body weight curves of the tumor-bearing mice after Lys/Arg dietary supplementation. The nine dietary supplementation regimens were generated from three Lys concentrations and three Arg concentrations. Each curve represents one Lys/Arg combination (*n* = 9–11). **c** 3D surface response analysis of body weight from day 3 to 22 based on the data shown in (**b**). The x- and y-axes indicate Lys and Arg concentrations, respectively, and the z-axis represents normalized body weight. Body weight data were normalized by min–max scaling. **d** Tumor volume curves of tumor-bearing mice after Lys/Arg supplementation. Tumor volume was calculated as 1/2 × L × W2, where L indicates the longest tumor diameter and W indicates the perpendicular shorter diameter. A maximum tumor volume of 2000 mm3 was used as the ethical endpoint (n = 9–11). **e** 3D surface response analysis of tumor volume from day 13 to day 22 based on the data shown in (**d**). Tumor volume data were normalized by min–max scaling to allow comparison across conditions. **f** Survival curve of tumor-bearing mice after Lys/Arg supplementation. Survival was monitored until animals reached the ethical endpoint or the end of observation (*n* = 7–11). The difference in sample size between body weight/tumor volume analyses and survival analysis was due to spleen collection for scRNA-seq. On day 19, three mice from the LLHA group and three from NLHA were sacrificed for spleen collection and were therefore excluded from the survival analysis, while their body weight and tumor volume data before day 19 were included in the corresponding analyses. **g** 3D surface response analysis of survival from day 15 to day 22 based on the data shown in (**f**). Data in (**c**) and (**e**) were normalized via min–max scaling using the formula: (individual value—minimum value)/(maximum—minimum value). The schematic in panel (**a**) was created by the authors using Adobe Illustrator. i.p., intraperitoneal injection; Lys, lysine; Arg, arginine

To evaluate the balance of Lys and Arg in tumor suppression in mammals, we conducted a dietary intervention study in B16F10 melanoma-bearing mice. Mice were initially maintained on a standard diet (0.9% Lys and 0.45% Arg). After subcutaneous injection of tumor cells, mice were assigned to Lys/Arg-free diets and administered with varying concentrations of Lys (0.0–0.50%) and Arg (0.50–2.0%) by intraperitoneal injection, informed by the optimal amino acid formulations identified in *Drosophila* (Fig. 2a). Surface response analysis revealed a dose-dependent interaction between Lys and Arg that significantly modulated body weight, tumor volume, and survival rates. To allow comparison across metrics, body weight and tumor volume data were normalized using the relative expression: values were calculated as (individual value-minimum value)/(maximum-minimum value). Also, we performed the analyses based on absolute body weight and tumor volume values.

The Lys-Arg balance had a marked effect on body weight. The optimal weight maintenance was achieved with diets containing 0.25–0.5% Lys and 0.5–1.0% Arg, with the combination of 0.5% Lys/0.5% Arg yielding the highest body weight (Fig. 2b-c, Fig. S2a-d). In contrast, the 0.0% Lys/2.0% Arg combination induced severe weight loss, highlighting the strongly adverse effects of extremely high Arg in the absence of Lys (Fig. 2b-c, Fig. S2a-d). The Lys-Arg balance also significantly impacted tumor growth. Diets with either 0% Lys or 2% Arg significantly suppressed tumor growth compared to other regimens (Fig. 2d, Fig. S3a-c). However, tumor suppression was weakest at intermediate Lys-Arg combinations (0.25–0.5% Lys/0.5–1% Arg), with the 0.25% Lys/1.0% Arg group displaying the highest tumor volume, indicating a tumor-promoting effect associated with this specific combination (Fig. 2e, Fig. S3d). Survival outcomes further emphasized the complexity of Lys-Arg interactions. High Arg (2.0%) correlated with poor survival, whereas moderate Arg levels (0.5–1.0%) improved outcomes (Fig. 2f, Fig. S4a-c). Diets combining low Lys (0.25–0.50%) and moderate Arg (0.5–1.0%) significantly enhanced survival, with the 0.5% Lys/0.5% Arg regimen consistently yielding the best overall outcomes (Fig. 2f-g, Fig. S4d). Thus, survival did not fully parallel tumor suppression, highlighting that the optimal regimen balances tumor control and host fitness: 0% Lys/1% Arg achieved the strongest tumor inhibition but compromised body weight, whereas 0.25% Lys/1% Arg permitted maximal tumor growth despite weight maintenance; in contrast, 0.5% Lys/0.5% Arg provided the most favorable overall outcome by improving survival while preserving body weight.

### Imbalance of Lys and Arg alters the equilibrium of immune cell populations

**Fig. 3:**
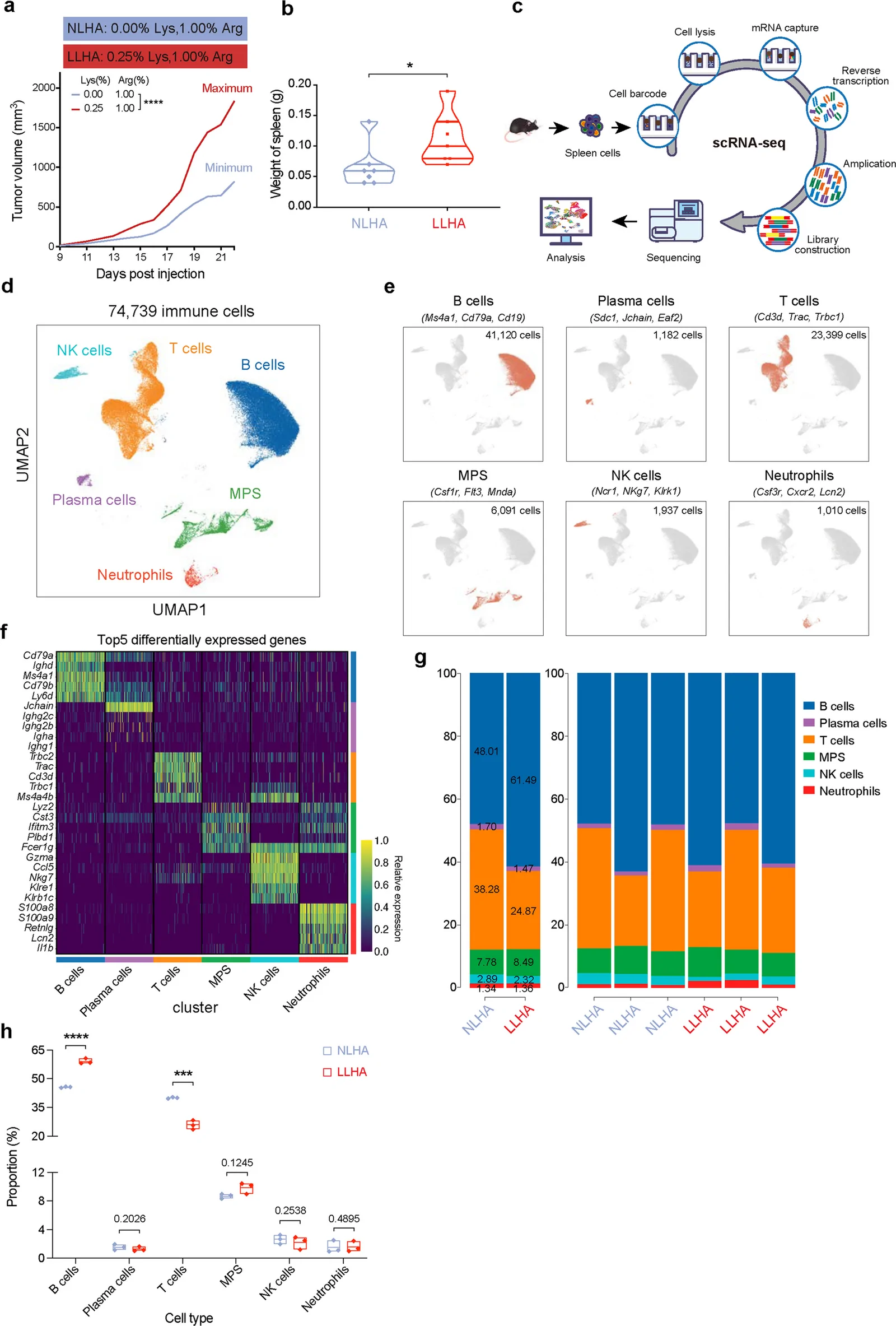
Imbalance of Lys and Arg alters the equilibrium of immune cell populations. **a** Tumor volume curves of B16F10 tumor-bearing mice receiving 0.00% Lys/1.00% Arg supplementation, referred to as Null Lys and High Arg (NLHA), or 0.25% Lys/1.00% Arg supplementation, referred to as Low Lys and High Arg (LLHA). Both two groups were selected for scRNA-seq because NLHA showed the strongest tumor suppression, whereas LLHA showed the weakest tumor inhibition and highest tumor volume (*n* = 9–11). **b** Weight of spleen (n = 7). **c** The workflow of spleen 10 × single-cell RNA sequencing. Spleen samples were collected from tumor-bearing mice fed NLHA or LLHA on day 19 post-tumor inoculation. Fresh spleens were processed into single-cell suspensions, followed by 10 × single-cell library preparation, sequencing, quality control, clustering, and cell-type annotation (*n* = 3). **d** UMAP visualization of 74,739 splenic immune cells from NLHA and LLHA groups after integration and clustering. Each dot represents one single cell. Colors indicate annotated major immune cell populations. **e** Feature plots showing expression of representative marker genes used to annotate major splenic immune cell populations, including B cells, plasma cells, T cells, mononuclear phagocyte system (MPS), NK cells, and neutrophils. **f** Heatmap showing the relative expression of the top five canonical marker genes across major splenic immune cell populations. Color intensity indicates scaled gene expression levels. **g** Stacked bar chart showing the proportions of major splenic immune cell populations in NLHA and LLHA groups. Each bar represents the cellular composition of one treatment group (*n* = 3). **h** Percentage of major spleen immune cell populations in the spleen after NLHA or LLHA supplementation. Each dot represents one mouse (*n* = 3). Data are presented as mean ± SEM. Tumor volume in (**a**) was analyzed using two-way ANOVA. Weight of spleen in (**b**) and cell-population proportions in (**h**) were analyzed using an unpaired two-tailed t-test. *p < 0.05; **p < 0.01; ***p < 0.001; ****p < 0.0001. UMAP, uniform manifold approximation and projection; scRNA-seq, single-cell RNA sequencing; NLHA, 0% Lys/1% Arg; LLHA, 0.25% Lys/1% Arg; MPS, mononuclear phagocyte system

The host immune response plays a critical role in controlling tumor progression [26]. To investigate how dietary manipulation of Lys and Arg affects immune cells, we focused on the spleen, which is the largest lymphatic organ and a key mediator of immune responses [27]. We compared the effects of two distinct diets: the combination of 0.25% Lys and 1% Arg (Low Lys and High Arg, LLHA), which showed the weakest inhibition of tumor growth, and 0% Lys and 1% Arg (Null Lys and High Arg, NLHA), which exhibited the strongest tumor growth suppression (Fig. 3a). LLHA significantly increased weight of spleen (Fig. 3b). To understand the mechanistic differences underlying these dietary effects, we constructed a comprehensive immune cell atlas from the splenic tissues of mice fed LLHA and NLHA. This was accomplished through high-resolution single-cell RNA sequencing (scRNA-seq). Fresh spleen tissues were processed into single-cell suspensions and subjected to droplet-based 10 × Chromium single-cell transcriptome profiling (Fig. 3c). After rigorous quality control and filtering, we obtained detailed gene expression profiles for 74,739 immune cells across these two dietary groups (Table S1). Gene expression normalization, followed by principal component analysis, allowed us to identify highly variable genes across the datasets. We then performed graph-based clustering to define distinct transcriptional subpopulations within the immune cell populations.

Single-cell RNA-seq clustering identified six major immune cell populations based on canonical markers: B cells (*Ms4a1*, *Cd79a*, *Cd19*), plasma cells (*Sdc1*, *Jchain*, *Eaf2*), T cells (*Cd3d*, *Trac*, *Trbc1*), mononuclear phagocyte system (MPS [28, 29]) (*Csf1r*, *Flt3*, *Mnda*), NK cells (*Ncr1, NKg7, Klrk1*) and neutrophils (*Csf3r*, *Cxcr2*, *Lcn2*), each characterized by unique transcriptional signatures (Fig. 3d-e). Using differential gene expression analysis, each immune cell type was cleanly defined by unique gene modules (Fig. 3f, Table S2). LLHA significantly altered immune cell composition (Fig. 3g-h). Compared to NLHA, LLHA significantly increased the proportion of B cells while reducing T cell abundance (Fig. 3g-h), suggesting a potential bias toward B cell expansion and T cell proliferation suppression. Additionally, LLHA also demonstrated a trend toward increase, albeit not significantly, in the proportion of MPS (Fig. 3h). Together, these results indicate that LLHA reprograms splenic immunity by expanding B cells and MPS, potentially favoring antibody-mediated responses, while reducing T cells, likely suppressing T cell-mediated activities defenses, highlighting a shift in immune strategy driven by dietary Lys and Arg imbalances.

### Imbalance of Lys and Arg dysregulates B cell development

**Fig. 4:**
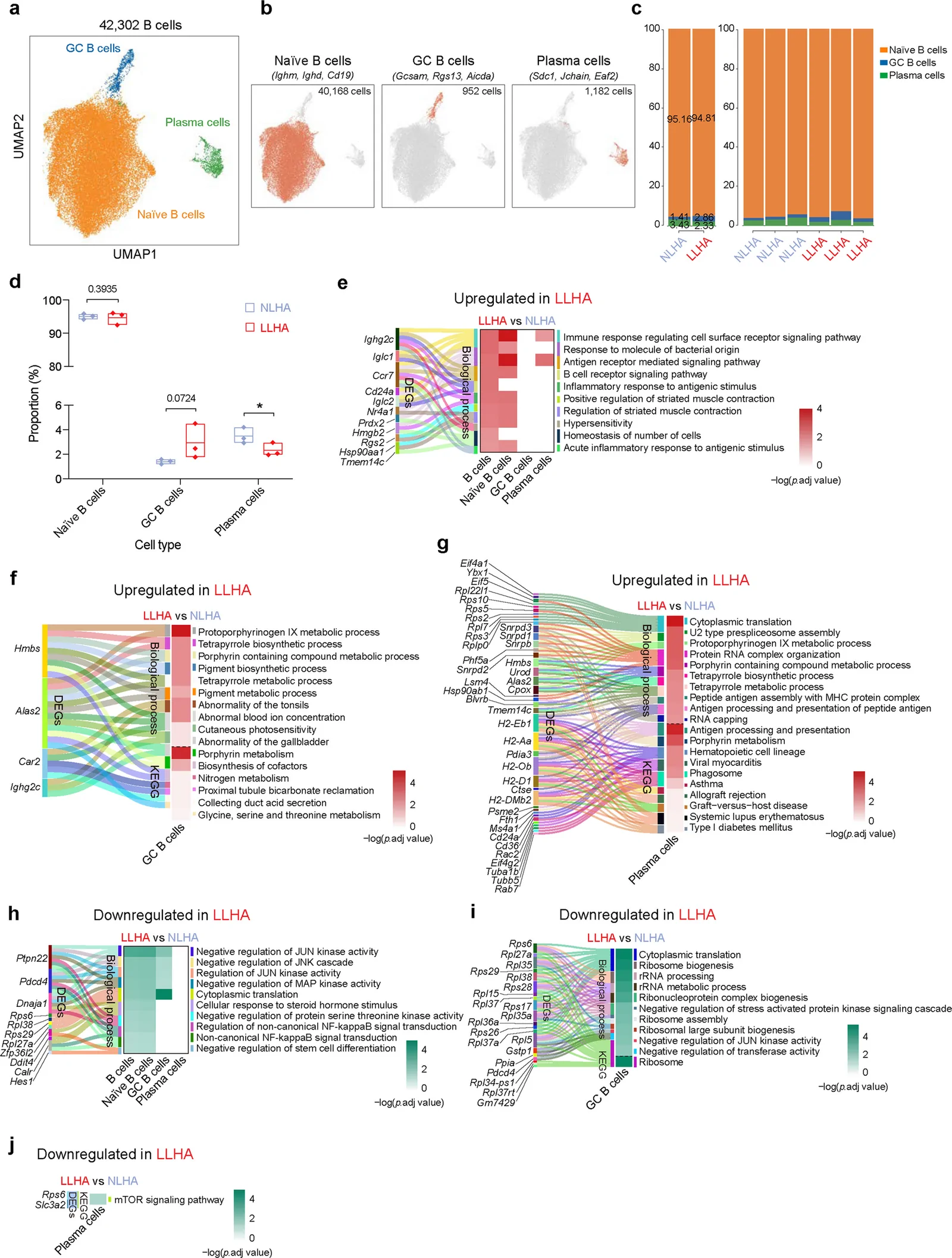
Imbalance of Lys and Arg dysregulates B cell development. **a** UMAP clustering of integrated B cells from NLHA and LLHA spleens. A total of 42,302 B cells were analyzed and annotated into three B-cell subpopulations. Each dot represents one single cell. Colors indicate B-cell subset identity. **b** Feature plots showing representative marker genes used for B-cell subset annotation. Naïve B cells were annotated using markers including *Ighm*, *Ighd*, and *Cd19*. Germinal center B cells were *Gcsam*, *Rgs13*, and *Aicda*. Plasma cells were *Sdc1*, *Jchain*, and *Eaf2*. **c** Stacked bar chart presenting the proportion of each B cell population after NLHA or LLHA supplement (*n* = 3). **d** Percentage of three B cell populations in the spleen after NLHA or LLHA supplementation. Each dot represents one mouse. (*n* = 3). **e**-**j** An integrated Sankey-heatmap visualization depicting the enrichment of biological processes and KEGG pathways among differentially expressed genes (DEGs) upregulated or downregulated in LLHA compared to NLHA. B cells and three B cell subsets (**e**, **h**), germinal center B cells (GC B cells) (**f**, **i**), and Plasma cells (**g**, **j**). The Sankey component links cell subsets to enriched pathways, and the heatmap component indicates pathway enrichment strength. Data are presented as mean ± SEM. Unpaired T test for (**d**). *p* values were adjusted using the Benjamini–Hochberg procedure. *p < 0.05. GC B cells, germinal center B cells; DEGs, differentially expressed genes; KEGG, Kyoto Encyclopedia of Genes and Genomes; NLHA, 0% Lys/1% Arg; LLHA, 0.25% Lys/1% Arg

We next investigated the Lys and Arg imbalance on B cells. Analysis of 42,302 B cells identified three major B cell populations: naïve B cells (*Ighm*, *Ighd*, *Cd19*), germinal center (GC) B cells (*Gcsam*, *Rgs13*, *Aicda*), and plasma cells (*Sdc1*, *Jchain*, *Eaf2*), each characterized by unique transcriptional signatures (Fig. 4a-b). Dietary manipulation with LLHA significantly altered B cell composition compared to NLHA (Fig. 4c). The proportion of GC B cells, critical for adaptive immunity via activation and differentiation, showed a nearly significant increase in LLHA-fed mice (Fig. 4d). In contrast, naïve B cells, which are antigen-inexperienced, remained unaffected by the LLHA diet (Fig. 4d). Interestingly, plasma cells, which are responsible for antibody production, were significantly reduced in LLHA-fed mice (Fig. 4d). This suggests that LLHA promotes GC B cell expansion but limits their terminal differentiation into plasma cells.

Notably, LLHA significantly altered gene expression in naïve B cells, and upregulated pathways associated with B cell signaling and antigen response, suggesting heightened naïve B cell activation upon antigen exposure (Fig. 4e). The upregulated pathways differed between GC B cells and plasma cells, with unique signatures in each subset. In GC B cells, pathways related to pigment and tetrapyrrole metabolism were upregulated (Fig. 4f). Plasma cells also exhibited upregulation of the tetrapyrrole metabolism pathway, alongside enhanced cytoplasmic translation and antigen processing and presentation (APP) (Fig. 4g). In contrast, LLHA downregulated NF-κB and JNK signaling in naïve B cells (Fig. 4h), suppressed ribosome biogenesis, translation, and JNK signaling in GC B cells (Fig. 4i), and showed reduced enrichment of an mTOR-related pathway in plasma cells (Fig. 4j). Together, these findings indicate that LLHA expanded B-cell populations but limited their terminal differentiation into plasma cells, indicating an incomplete humoral response that may contribute to weak tumor inhibition; consistently, *Rps6*, the downstream component of mTORC1 signaling and translational regulation, was significantly downregulated under LLHA conditions (Fig. S5a).

### Imbalance of Lys and Arg dysregulates T-cell development

**Fig. 5:**
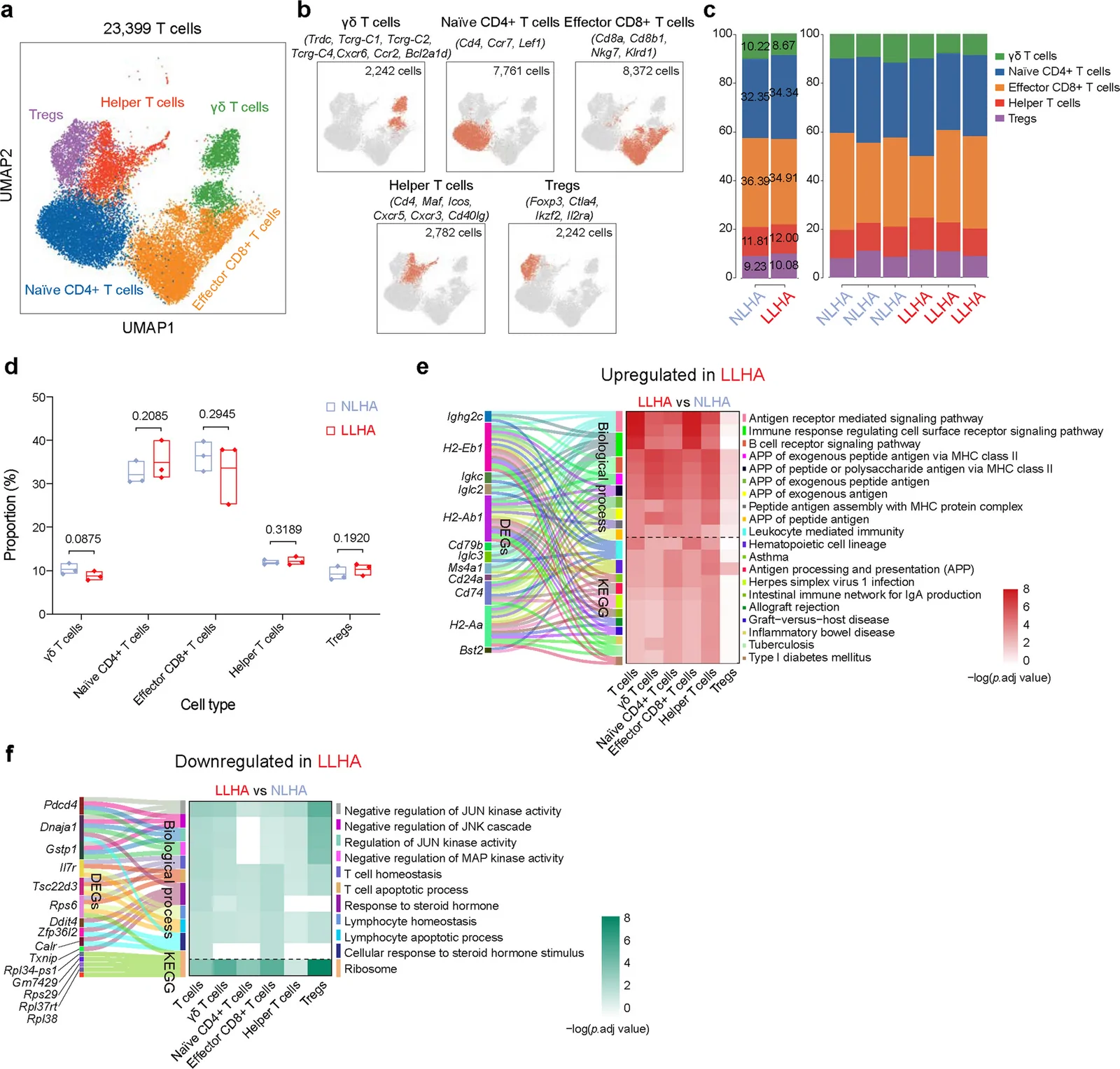
Imbalance of Lys and Arg dysregulates T-cell development. **a** UMAP clustering of integrated T cells from NLHA and LLHA spleens. A total of 23,399 T cells were analyzed and annotated into five T-cell subsets. Each dot represents one single cell. Colors indicate T-cell subset identity. **b** Feature plots showing representative marker genes used for T-cell subset annotation. γδ T cells were annotated using markers including *Trdc*, *Tcrg-C1*, *Tcrg-C2*, *Tcrg-C4*, *Cxcr6*, *Ccr2*, and *Bcl2a1d*. Naïve CD4^+^ T cells were annotated using *Cd4*, *Ccr7*, and *Lef1*. Effector CD8^+^ T cells were annotated using *Cd8a*, *Cd8b1*, *Nkg7*, and *Klrd1*. Helper T cells were annotated using *Cd4*, *Maf*, *Icos*, *Cxcr5*, *Cxcr3*, and *Cd40lg*. Tregs were annotated using *Foxp3*, *Ctla4*, *Ikzf2*, and *Il2ra*. **c** Stacked bar chart showing the proportions of T-cell subsets in NLHA and LLHA groups. (*n* = 3). **d** Percentage of five T cell populations in the spleen after NLHA or LLHA supplementation. Each dot represents one mouse (*n* = 3). **e**–**f** Integrated Sankey-heatmap visualization showing enriched biological processes and KEGG pathways among DEGs in LLHA compared with NLHA. Panel (**e**) shows pathways enriched among upregulated genes in total T cells and five T-cell subsets, and panel (**f**) shows pathways enriched among downregulated genes. The visualization links each T-cell subset to the corresponding enriched biological processes or KEGG pathways. Data are presented as mean ± SEM in (**d**). Unpaired two-tailed t-test was used for (**d**). Pathway enrichment *p* values were adjusted using the Benjamini–Hochberg procedure. APP, antigen processing and presentation; DEGs, differentially expressed genes; KEGG, Kyoto Encyclopedia of Genes and Genomes; Tregs, regulatory T cells; NLHA, 0% Lys/1% Arg; LLHA, 0.25% Lys/1% Arg

Transcriptional cluster characterization identified five distinct T cell subsets: γδ T cells (*Trdc*, *Tcrg-C1*, *Tcrg-C2*, *Tcrg-C4*, *Cxcr6*, *Ccr2*, *Bcl2a1d*), naïve CD4^+^ cells (*Cd4, Ccr7, Lef1*), effector CD8^+^ T cells (CD8 Teff) (*Cd8a*, *Cd8b1*, *Nkg7*, *Klrd1*), helper T cells (Ths) (*Cd4*, *Maf*, *Icos*, *Cxcr5*, *Cxcr3*, *Cd40lg*), and regulatory T cells (Tregs) (*Foxp3*, *Ctla4*, *Ikzf2*, *Il2ra*) (Fig. 5a-b). Compared to the NLHA, LLHA did not significantly alter the proportion of T cell subsets (Fig. 5c-d). However, LLHA showed a suggestive decrease trend in γδ T cells. The reduction in γδ T cells and alterations in proliferating T cells suggest a potential vulnerability in early tumor detection.

In almost all T cell subsets, LLHA upregulated pathways associated with antigen/B cell receptor signaling, APP, and leukocyte-mediated immunity (Fig. 5e). In contrast, it downregulated genes involved in JNK signaling, T cell homeostasis, and apoptosis (Fig. 5f). This may partially explain the reduced populations of T cells observed in LLHA-fed mice. These changes suggest that LLHA promotes T cell activation but impairs terminal differentiation, potentially compromising effector functions. Together, these findings (Figs. 3g and 5) demonstrate that LLHA decreases the number of T cells (Fig. 3g) through the suppression of T cell proliferation.

### Imbalance of Lys and Arg differentially reprograms MPS functionality

**Fig. 6:**
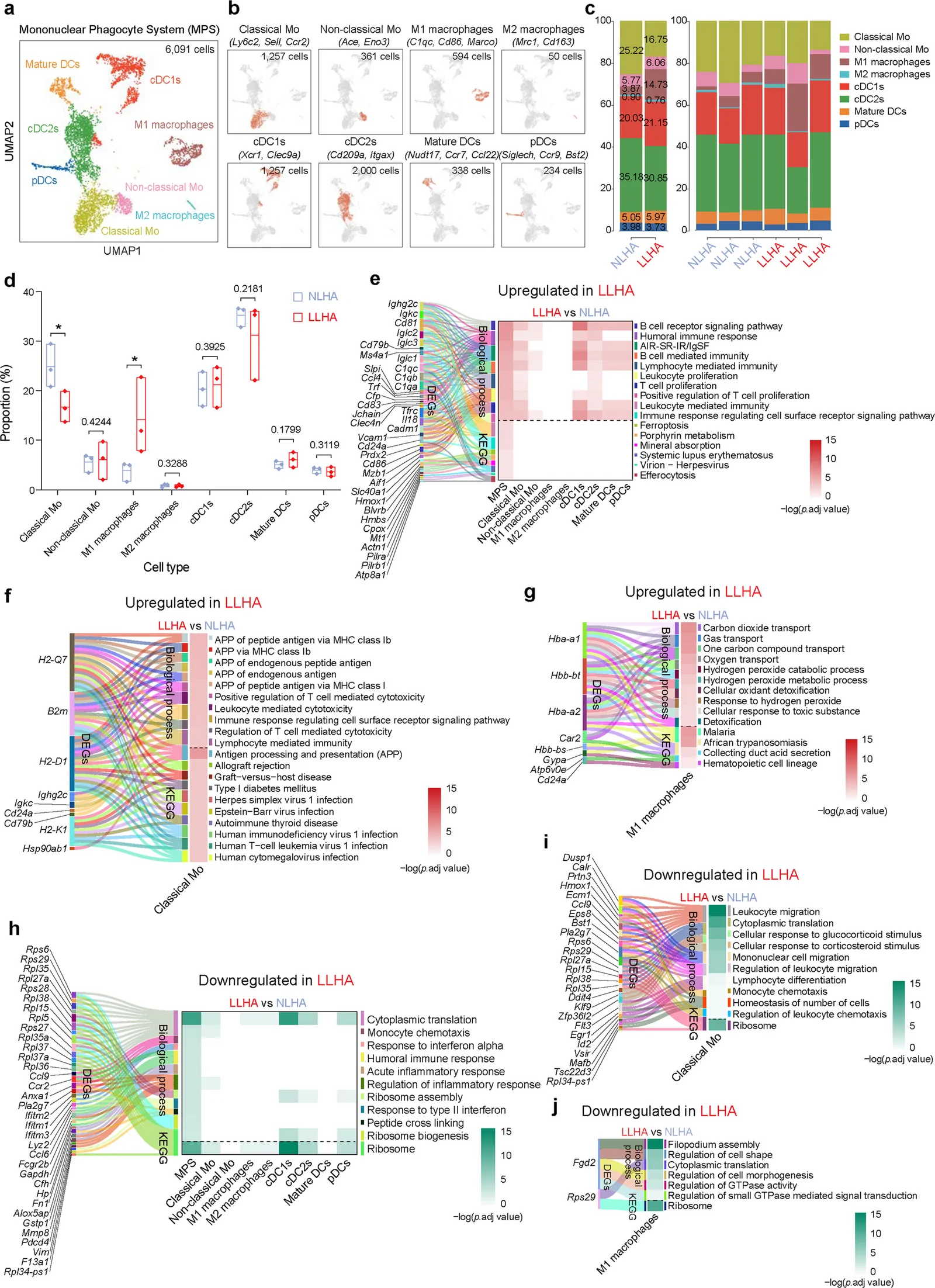
Imbalance of Lys and Arg differentially reprograms MPS functionality. **a** UMAP clustering of integrated mononuclear phagocyte system (MPS) cells from NLHA and LLHA spleens. A total of 6,091 MPS cells were analyzed and annotated into eight MPS subsets. Each dot represents one single cell. Colors indicate MPS subset identity. **b** Feature plots showing representative marker genes used for MPS subset annotation. Classical monocytes were annotated using *Ly6c2*, *Sell*, and *Ccr2*. Non-classical monocytes were annotated using *Ace* and *Eno3*. M1 macrophages were annotated using a combined marker-gene pattern including *C1qc*, *Cd86*, and *Marco*. M2 macrophages were annotated using *Mrc1* and *Cd163*. cDC1s were annotated using *Xcr1* and *Clec9a*. cDC2s were annotated using *Cd209a* and *Itgax*. Mature dendritic cells were annotated using *Nudt17*, *Ccr7*, and *Ccl22*. pDCs were annotated using *Siglech*, *Ccr9*, and *Bst2*. **c** Stacked bar chart showing the proportions of MPS subsets in NLHA and LLHA groups. (*n* = 3). **d** Quantification of eight MPS populations in the spleen after NLHA or LLHA supplementation. Each dot represents one mouse (*n* = 3). **e**-**j** An integrated Sankey-heatmap visualization depicting the enrichment of biological processes and KEGG pathways among differentially expressed genes (DEGs) upregulated or downregulated in LLHA compared to NLHA. MPS and eight MPS subsets (**e**, **h**), Classical Mo (**f**, **i**), M1 Macrophages (**g**, **j**). Data are presented as mean ± SEM in (**d**) and (**k**-**m**). Unpaired two-tailed t-test was used for cell-proportion analysis and qPCR comparisons. Pathway enrichment *p* values were adjusted using the Benjamini–Hochberg procedure. *p < 0.05. MPS, mononuclear phagocyte system; Classical Mo, classical monocytes; Non-classical Mo, non-classical monocytes; cDC1s, conventional dendritic cell type 1; cDC2s, conventional dendritic cell type 2; pDCs, plasmacytoid dendritic cells; AIR-SR-IR/IgSF, adaptive immune response-somatic recombination of immune receptors/immunoglobulin superfamily; APP, antigen processing and presentation; DEGs, differentially expressed genes; KEGG, Kyoto Encyclopedia of Genes and Genomes; NLHA, 0% Lys/1% Arg; LLHA, 0.25% Lys/1% Arg

Further exploration of the immune landscape identified eight distinct MPS subsets: classical monocytes (Classical Mo) (*Ly6c2*, *Sell*, *Ccr2*), non-classical monocytes (Non-classical Mo) (*Ace*, *Eno3*), M1 macrophages (*C1qc*, *Cd86*, *Marco*), M2 macrophages (*Mrc1*, *Cd163*), conventional dendritic cell type 1 (cDC1s) (*Xcr1*, *Clec9a*), conventional dendritic cell type 2 (cDC2s) (*Cd209a*, *Itgax*), mature dendritic cells (Mature DCs) (*Nudt17*, *Ccr7*, *Ccl22*), and plasmacytoid dendritic cells (pDCs) (*Siglech*, *Ccr9*, *Bst2*) (Fig. 6a-b). Most of the MPS subsets remained unaffected by LLHA when compared to NLHA (Fig. 6c-d). However, LLHA significantly decreased classical monocytes while significantly increasing M1 macrophages relative to NLHA.

LLHA upregulated pathways linked to B cell signaling, immunity, and T cell proliferation in most subsets of MPS (Fig. 6e). In classical Mo, LLHA enhanced pathways critical for APP and T cell-mediated cytotoxicity (Fig. 6f). In M1 macrophages, LLHA elevated pathways involved in carbon dioxide/oxygen transport and hydrogen peroxide metabolism (Fig. 6g). Conversely, LLHA-fed mice exhibited fewer top-downregulated pathways overall (Fig. 6h). In classical monocytes, LLHA suppressed pathways involved in cell migration, cytoplasmic translation, corticosteroid response, and ribosome biogenesis, indicating reduced migration and protein synthesis (Fig. 6i). In M1 macrophages, LLHA downregulated pathways associated with filopodium assembly, cell morphogenesis, GTPase activity, protein translation, and ribosome function (Fig. 6j). In conclusion, LLHA reduces translational activity while promoting the differentiation of M1 macrophages. qPCR validation confirmed that LLHA significantly increased *Hba-a1* and *Hbb-bt* expression (Fig. S5b–c, consistent with the hemoglobin pattern in Fig. 6g) and decreased *Rps29* (Fig. S5d, consistent with the ribosomal/translational signature in Fig. 6j), supporting LLHA-associated transcriptional remodeling in M1 macrophages and suggesting that Lys/Arg imbalance may alter MPS functionality and macrophage contributions to tumor immunity and inflammation.

## Discussion

Our cross-species investigation uncovers conserved mechanisms by which Lys and Arg interact to influence tumor suppression and host physiology. In *Drosophila*, survival is optimized at 0–0.5% Lys (except 0.25%) combined with 1.0% Arg. In mice, a balanced regimen of 0.5% Lys and 0.5% Arg maximizes survival and host fitness. Interestingly, a regimen of 0% Lys with 1% Arg achieves maximal tumor suppression but induces severe weight loss, highlighting a trade-off between therapeutic efficacy and systemic health. Conversely, the combination of 0.25% Lys and 1.0% Arg maximizes tumor growth (Fig. 2e) despite sustaining body weight (Fig. 2b), revealing a decoupling between host weight maintenance and tumor suppression. Notably, 0.25% Lys was also found to be detrimental to the survival of Drosophila (Fig. 1e). Furthermore, scRNA-seq reveals that the specific Lys/Arg formulation disrupts immune equilibrium, skewing splenic lymphocyte populations toward B cell expansion and depleting cytotoxic T cells, an immune shift strongly correlated with increased tumor volume. This suggests that tumor progression may be facilitated by diet-driven immunosuppression. Our findings provide a preclinical framework for exploring how species-specific thresholds and immune-nutritional interactions may inform future precision-nutrition strategies.

Current dietary interventions for cancer predominantly focus on isolated Lys restriction or Arg supplementation [13, 30]. While Lys restriction inhibits tumor growth in preclinical models [19] and exerts antiproliferative effects across cancer cell lines [21], clinical studies of Arg modulation in either deprivation or supplementation show inconsistent therapeutic outcomes [31–33]. This discrepancy likely stems from Arg’s dual roles in tumor biology [34]: moderate supplementation enhances immune function and cancer surveillance [35] meanwhile promotes tumor progression, whereas excessive Arg may promote tumor progression. We demonstrate that Arg exhibits a bell-shaped dose–response relationship across species. In *Drosophila*, 1% Arg optimally enhances survival, whereas in murine models, the same concentration maximizes tumor progression. These findings highlight the strong concentration-dependent effects of Arg, necessitating precise control in dietary interventions. In contrast, the effects of Lys are more complex: while lower Lys concentrations (below 0.5%) generally correlate with improved outcomes, our data demonstrate a paradox at 0.25% Lys. *Drosophila* and mouse findings identify 0.25% Lys as an empirically sensitive threshold. LLHA downregulated *Rps6*, consistent with the mTOR-related plasma-cell transcriptional signature, suggesting altered mTOR-related translational programs; however, direct mTOR activity was not assessed. Unchanged *Slc7a1* expression argues against a transcriptional driver (Fig. S6); future isotope-uptake, transporter-inhibition, or SLC7A1-perturbation studies are required to test whether Lys/Arg competition at the transporter level contributes to tumor and immune outcomes. Collectively, these results indicate that Lys interventions should avoid the 0.25% concentration range to prevent adverse biological consequences.

Our systematic investigation of dosage combinations reveals that antitumor efficacy strictly depends on a precise Lys-Arg balance. The regimen of 0.5% Lys (approximately 50% of normal) with 0.5% Arg (approximately normal level) optimally preserves host physiology, promotes weight gain, and prolongs survival in tumor-bearing mice. In contrast, the combination of 0.25% Lys with 1.0% Arg induces paradoxical tumor expansion despite metabolic stability. Meanwhile, complete Lys restriction (0%) with 1% Arg suppresses tumor growth but severely disrupts body weight maintenance, resembling cachexia. In addition, 2% Arg can strongly suppress tumor growth. Our study suggests that high concentrations of Arg (2%) may modulate the immune microenvironment in ways that enhance immune surveillance. While 1% Arg in mouse model promoted tumor progression, the 2% concentration appears to skew the immune response toward a more potent antitumor profile. This shift could involve enhanced macrophage polarization and T cell activation, which could contribute to stronger tumor suppression despite the higher tumor metabolic demands. However, it is crucial to note that the exact mechanisms behind this effect warrant further investigation to fully understand how excessive Arg influences immune cell function and its balance with tumor biology. All these indicate that effective tumor suppression requires balanced amino acid modulation rather than maximal single-nutrient restriction. Our data reconcile contradictory findings in single-agent studies by emphasizing the importance of amino acids crosstalk. Therefore, the therapeutic efficacy of Lys/Arg modulation in cancer should consider the combined effects of Lys and Arg to achieve favorable antitumor responses without compromising host health.

In this study, we unveil a compelling ​nutrient-immunity paradox​ in cancer biology. Moderate Lys restriction (0.25%) amplifies innate immunity (M1 macrophages) and expands B cell populations, yet insufficiency of essential Lys critically undermines B cell functional maturation, a process demanding robust nutrient support [36–38]. This suggests a possible immune-metabolic imbalance induced by disrupted Lys/Arg balance, with LLHA representing a pronounced example in which altered systemic immune remodeling may create a permissive condition for tumor progression. Conversely, 0.5% Lys establishes a delicate equilibrium, providing sufficient resources to sustain both innate and adaptive immunity, thereby tipping the balance toward tumor suppression and survival. The contrast between 0% Lys (minimal tumors but attenuated fitness) and 0.25% Lys (hyper-progressive tumors) underscores that ​Lys restriction alone cannot sustainably combat cancer. Immune cells themselves require a "therapeutic window" of essential amino acids to function effectively. This aligns with our observation that Arg, while pro-immunogenic, exhibits a narrow safe dose range: excessive restriction harms immunity while excess may fuel tumor anabolism.

While Lys sufficiency sustains adaptive immunity [39–41], our study reveals a paradoxical immune-metabolic crosstalk when combining 0.25% Lys with 1% Arg. Although Arg supplementation typically enhances T cell immunity [24, 42–44], this specific combination induced the suppression of T cell homeostasis and differentiation, thereby crippling cell-mediated antitumor responses. Simultaneously, B cell hyperproliferation coupled with plasma cell differentiation blockade generated non-functional humoral immunity. This dual immunologic sabotage with the exhaustion of cellular defenses and production of immature antibody effectors creates an "immune vacuum" that accelerates tumor growth. Furthermore, this combination induced an expansion of M1 macrophages in the spleen, indicative of a heightened systemic inflammatory response that paradoxically fueled tumor progression [45]. The increase in splenic M1 macrophages may not directly indicate their infiltration into the tumor microenvironment; thus, their cytotoxic role at the tumor site remains uncertain [46–48]. Our data challenge the conventional approach of single amino acid modulation in nutritional oncology, demonstrating that amino acid combinations determine antitumor immune competence.

Several limitations should be considered. First, the cross-species convergence may be confounded by absolute dose, Lys/Arg ratio, route, duration, sex, age, and species-specific metabolism; because lysine is strictly exogenous while arginine is endogenously synthesized and recycled, identical dietary percentages do not imply equivalent bioavailability. Accordingly, direct measurement of plasma Lys and Arg levels will be necessary in future studies. Our findings therefore represent a preclinical response map, not a translatable prescription. Second, the *Drosophila* RasV12-GFP and mouse B16F10 melanoma models limit generalizability. The immunogenic B16F10 tumor may not reflect responses in poorly immunogenic or metabolically distinct cancers; broader testing across lung, pancreatic, and colorectal models is needed. Third, splenic profiling captures host-level immune remodeling rather than the tumor microenvironment; tumor-tissue analyses are required to determine whether splenic changes are mirrored locally. Moreover, scRNA-seq immune associations do not establish causality-functional experiments (depletion, reprogramming, rescue) must test whether specific immune shifts are necessary or sufficient for tumor acceleration. Fourth, Lys/Arg modulation likely alters systemic metabolism beyond tumor and spleen; body weight and survival are inadequate surrogates. Future studies should assess circulating amino acids, glucose-insulin homeostasis, lipid profiles, hepatic/renal function, and endocrine mediators such as FGF21, given that amino-acid restriction engages mTORC1, GCN2, and the integrated stress response [49–51] with potential impact on tumor and host physiology [13]. Finally, the preclinical percentages are not human prescriptions: a mouse receiving ~15–25 mg Lys/day contrasts with adult requirements (~30 mg/kg/day) and habitual arginine intake (~4–5 g/day) [52–55]. Although modulation is physiologically feasible, clinical translation requires controlled formulation, plasma amino-acid monitoring, immune profiling, and safety evaluation.

## Materials and methods

### Animals

*Drosophila melanogaster* strain w^1118^ were reared and cultivated in a controlled environmental chamber (PQX-600A-12HM, Lefebvre Technology, Ningbo), maintained at a temperature of 25 °C and 60% relative humidity with a 12-h light/dark cycle [56]. All fly lines utilized in this study were devoid of Wolbachia endosymbionts.

Seven week-aged female C57BL/6 J mice were procured from Vital River Laboratory Animal Technology Co., Ltd. (Zhejiang, China). The animals were maintained in a specific pathogen-free environment at a temperature of 23 ± 2 °C, with a relative humidity of 50—65%, and under a standard light/dark cycle of 12 h each.

### Cell culture

The oncogenic RasV12 cell line, established by A. Simcox et al. in 2008 [57], incorporates a green fluorescent protein (GFP) marker, thereby enabling the quantification of cell proliferation through the measurement of GFP expression levels. These RasV12-GFP cells were cultured in Schneider's *Drosophila* Medium (Gibco), enriched with 10% Fetal Bovine Serum (FBS, Gibco), 1% Glutamax (Invitrogen), 1% Penicillin/Streptomycin (HyClone), and 500 μg/mL Puromycin (Invitrogen) to maintain optimal growth conditions [56].

The B16F10 cell line was donated by the laboratory of Professor Zhang Xudong [58], School of Medicine at Sun Yat-sen University. The cells were cultured in Dulbecco's Modified Eagle's Medium (DMEM; Gibco), which was supplemented with 10% FBS (Gibco) and 1% Penicillin/Streptomycin (HyClone).

### Injection of RasV12-GFP oncogenic cells into flies and measurement

Post-harvest, GFP-positive cells in PBS were quantified using the Countess II FL Automated Cell Counter (Thermo Fisher Scientific). A precise quantity of 500 RasV12-GFP cells were injected into the thorax of 3–5-day-old adult flies, which had been anesthetized using CO_2_, at a volume of 10 nL per fly. This procedure was executed with a programmable nanoliter injector (Nanoject III™; Drummond Scientific). The tumor-bearing flies were reared at 25 °C and provided with basal diets or normal diets. The survival rate of the tumor-bearing flies was assessed daily until their demise, with two biological replicates for each time point, each consisting of 60 flies. Flies injected with the same volume of PBS were subjected to the above same procedure as the control group.

### Tumor-bearing fly feeding experiments

Prior to the injection of RasV12-GFP oncogenic cells, the flies were nourished on a standard yeast-maize medium. Post-injection with RasV12-GFP cells, adult male flies aged 3–5 days were categorized into 36 distinct treatment cohorts and allocated to 36 unique dietary regimens. We formulated 36 diets with varying compositions of lysine (Lys) and arginine (Arg) by incorporating six distinct concentrations of Lys (0%, 0.125%, 0.25%, 0.5%, 1%, 2%) and Arg (0%, 0.125%, 0.25%, 0.5%, 1%, 2%) into a basal diet comprising 1% yeast (product #CY0399, Angel Yeast) and 5% sucrose (product #57–50-1, Guangzhou Sugar). Each experimental replicate involved a minimum of 60 flies per group, with a minimum of two replicates conducted for each condition.

### CDX model establish of subcutaneous melanoma in mouse

Following a one-week acclimatization period, an 8-week-old female mouse (about 17 g) for subcutaneous melanoma was induced by subcutaneously injecting 6 × 10^4^ B16F10 cells suspended in 100 μL PBS. Tumor dimensions were assessed daily from day 9 post-inoculation using the established tumor size formula [59]: tumor volume: 1/2 × L × W^2^, where *L* represents the longest dimension, and W is the width, measured as the shorter dimension perpendicular to L and parallel to the mouse's body.

### Tumor-bearing mouse feeding experiments

A basal diet devoid of Lys and Arg, with other ingredients aligning with the AIN-93 M standard diet specifications, was introduced from the second day post-construction of the subcutaneous melanoma (B16F10 cells) model. Mice were stratified into nine groups based on body weight to ensure equal average weights per group (~17 g). The calculated total daily food intake per mouse was about 3 g [60], with an intraperitoneal injection volume of 0.4 mL; PBS served as the blanking solvent administered once daily. The final daily supplementation levels of Lys or Arg at 0%, 0.25%, 0.5%, 1%, and 2% were equivalent to concentrations of 0 mg/mL, 18.75 mg/mL, 37.5 mg/mL, 75 mg/mL, and 150 mg/mL in PBS, respectively. The experimental design included nine groups with varying concentrations of Lys (0.0, 0.25, 0.5%) and Arg (0.5%, 1.0%, 2.0%). The overall welfare of the animals was monitored daily, with tumor volume, body weight and survival rate being recorded daily.

### Single-cell RNA sequencing and data analysis

On day 19 post-tumor modeling, fresh spleen samples from mice that had been fed diets with NLHA (0% Lys/1% Arg) and LLHA (0.25% Lys/1% Arg) were promptly preserved in sCelLive™ Tissue Preservation Solution (Singleron) on ice within 30 min of surgical extraction. The harvested specimens were rinsed with Hanks Balanced Salt Solution (HBSS) three times, finely minced, and subsequently subjected to enzymatic digestion using 3 mL of sCelLive™ Tissue Dissociation Solution (Singleron) via the Singleron PythoN™ Tissue Dissociation System at 37 °C for 15 min. The resulting cell suspension was collected and strained through a 40-micron sterile filter. Subsequently, GEXSCOPE® red blood cell lysis buffer (RCLB, Singleron) was introduced, and the mixture, prepared at a volume ratio of cells to RCLB of 1:2, was incubated at room temperature for 5–8 min to lyse red blood cells. The mixture was then centrifuged at 300 g at 4 °C for 5 min to pellet the cells and remove the supernatant. The cell pellet was gently resuspended in Phosphate-Buffered Saline (PBS). Ultimately, the samples were stained with Trypan Blue, and cell viability was assessed microscopically.

### Reverse transcription, cDNA amplification, and library preparation

Single-cell suspensions were adjusted to 2 × 10^5^ cells/mL in PBS and loaded onto micro-well chips with the Singleron Matrix® Single Cell Processing System. Following cell capture, barcoding beads were collected from the chips, and bead-bound mRNA was converted into cDNA by reverse transcription. The resulting cDNA was amplified by PCR and subsequently fragmented before sequencing adapters were ligated. Libraries were prepared using the GEXSCOPE® Single Cell RNA Library Kits (Singleron) according to the manufacturer's protocol. After quantification, individual libraries were normalized to 4 nM, pooled, and sequenced on an Illumina NovaSeq 6000 platform using 150-bp paired-end sequencing.

### Primary analysis of raw read data (scRNA-seq)

Raw sequencing reads were subjected to processing to generate gene expression profiles utilizing CeleScope (Singleron Biotechnologies) with the application of default parameters. The procedure commenced with the extraction and correction of barcodes and unique molecular identifiers (UMIs) from the R1 reads. Subsequently, adapter sequences and poly-A tails were excised from the R2 reads. The processed R2 reads were aligned to the GRCm38 (mm10) transcriptome using the STAR [61] aligner. Reads that mapped uniquely were then assigned to exons employing FeatureCounts [62]. Reads that were successfully assigned, sharing the same cell barcode, UMI, and gene identifier, were aggregated to construct the gene expression matrix, which was subsequently used for further downstream analysis.

### Quality control, dimension-reduction and clustering (Scanpy)

Scanpy was employed for quality control, dimensionality reduction, and clustering, executed within a Python 3.7 environment [63]. For each sample dataset, the expression matrix underwent a series of filtering criteria: 1) exclusion of cells with a gene count below 200 or those comprising the top 2% of gene counts; 2) exclusion of cells with UMI counts in the top 2%; 3) exclusion of cells with mitochondrial content exceeding 10%; 4) exclusion of genes detected in fewer than 5 cells. Post-filtering, 74,739 cells were selected for subsequent analyses, averaging 1,036 genes and 3,072 UMIs per cell. The raw count matrix was normalized based on the total counts per cell and subjected to logarithmic transformation to generate a normalized data matrix. The top 2,000 variable genes were identified by setting the flavor parameter to 'seurat' [64]. Principal Component Analysis (PCA) was conducted on the scaled variable gene matrix, leveraging the top 20 principal components for clustering and dimensionality reduction. Cellular clustering was performed using the Louvain algorithm with a resolution parameter set to 1.2, resulting in the segregation of cells into 25 distinct raw clusters. The distribution of cell clusters was visualized employing Uniform Manifold Approximation and Projection (UMAP).

### Batch effect removal

Batch effects between samples were mitigated through the application of Harmony [65], a computational method implemented in harmony. This process leveraged the top 20 principal components derived from PCA to harmonize the datasets, thereby reducing inter-sample variability attributable to technical sources rather than biological differences [66].

### Differentially expressed genes (DEGs) analysis (scanpy)

Differential gene expression analysis was conducted to discern differentially expressed genes (DEGs) utilizing the Scanpy [63] function scanpy.tl.rank_genes_groups. This method employed the Wilcoxon rank sum test with default parameters to statistically assess the expression differences. Genes were considered DEGs if they were expressed in more than 10% of the cells within at least one of the compared cellular groups and demonstrated an average logarithmic (base 2) fold change value exceeding 1. To control for multiple hypothesis testing, the *p* values were adjusted using the Benjamini–Hochberg procedure, with a threshold of 0.05 applied to determine statistical significance.

### Pathway enrichment analysis

To investigate the potential functions of DEGs between LLHA and NLHA, biological process (BP) and Kyoto Encyclopedia of Genes and Genomes (KEGG) pathway analyses were performed using the 'clusterProfiler' R package [67], version 4.2. Package ‘MSigDB’ was used for BP analysis. Pathways with an adjusted *p* value (*p*_adj) of less than 0.05 were deemed to be significantly enriched. The selected enriched pathways were visualized as heat plots to represent the findings.

### Quantitative real‑time PCR

The level of representative gene expression in spleen tissue, including *Rps6*, *Hba-a1*, *Hbb-bt*, *Rps29* and *Slc7a1*, was analyzed by qRT-PCR with primer sequences listed in Table S3. *Hprt1* was as an internal reference gene.

### Statistical analysis

Data were presented as mean ± SEM for each experimental group. Two-tailed unpaired T test one-way and two-way analysis of variance (ANOVA) were used to assess the statistical significance between two means. The log-rank test was performed for Kaplan–Meier survival analysis. The Wilcoxon rank sum test was employed for statistically assessing gene expression differences. All statistical analyses were performed using GraphPad Prism 9 software and R language. The choice of analytical methods was dictated by the design of each experiment, and specific details were provided in the corresponding figure legends. *p* < 0.05 was considered statistically significant. Statistical significance denoted as follows: n.s., not significant; *p < 0.05; **p < 0.01; ***p < 0.001, ****p < 0.0001.

## Supplementary Information

Supplementary Material 1.

Supplementary Material 2.

Supplementary Material 3.

Supplementary Material 4.

## Data Availability

This manuscript does not encompass any original computational code. However, all supplementary information required to perform an independent reanalysis of the data presented in this paper is accessible through the corresponding author upon request. The raw sequence data reported in this paper have been deposited in the Genome Sequence Archive [68] in the National Genomics Data Center [69], China National Center for Bioinformation /Beijing Institute of Genomics, Chinese Academy of Sciences (GSA: CRA025780) that are publicly accessible at https://ngdc.cncb.ac.cn/gsa. This study did not generate new unique reagents. All data and materials that support the findings of this study are available within the article and supplemental information or available from the lead contact upon request.
