# Supplementary material for: Fine-tuning the lysine-arginine duet: a dietary approach to suppress tumor growth
Source: Mol Biomed. 2026 Sep 22;7:182. doi: 10.1186/s43556-026-00588-0 (PMC13598008; doi:10.1186/s43556-026-00588-0)
Supplement: Supplementary file 1 [file 43556_2026_588_MOESM1_ESM.docx]

**Supplementary information**

**Fine-Tuning the Lysine-Arginine Duet: A Dietary Approach to Suppress Tumor Growth**

Ben-Chi Zhao, Xin-Yuan Zhao, Wen-Xiu Li, Xiao Lan, Xiao-Ming Huang, Hao Feng, An-Qi Li, Run-Lu Shi, Hui-Lin Li, Xi Xie, Guo-Hui Wan, Ren-Jie Jiao, Di Chen*, Jian-Sheng Kang*, Qiao-Ping Wang*

**Fig. S1** Optimal combination of Lys and Arg enhances survival in tumor-bearing *Drosophila*, related to Figure 1.

Lifespan of flies fed on a basal diet or normal diet with injected RasV12-GFP oncogenic cells or PBS (*n* = 21-237). The basal diet includes 1% yeast, 5% sucrose, and 1% agar. The normal diet includes 3.22% yeast, 3.16% sucrose, 6.32% glucose, 7.77% corn powder, 0.07% CaCl_2,_ and 1% agar.

A log-rank (Mantel-Cox) test was used for statistical analysis: n.s., statistically nonsignificant; ***p* < 0.01.

**Fig. S2** Balancing Lys and Arg optimize survival in tumor-bearing mice, related to Figure 2.

(**a**-**c**) Body weight curves across nine dietary supplement groups with graded Lys-Arg combinations from day 0 to day 22 (n = 9-11). (**a**): 0.00% Lys/0.50% Arg, 0.25% Lys/0.50% Arg, 0.50% Lys/0.50% Arg. (**b**): 0.00% Lys/1.00% Arg, 0.25% Lys/1.00% Arg, 0.50% Lys/1.00% Arg. (**c**): 0.00% Lys/2.00% Arg, 0.25% Lys/2.00% Arg, 0.50% Lys/2.00% Arg.

(**d**) 3D surface response analysis of body weight from day 3 to 22 based on the data shown in (**a**-**c**). The x- and y-axes indicate Lys and Arg concentrations, respectively, and the z-axis represents body weight.

Data were reported as mean ± SEM. Two-way ANOVA with Sidak multiple comparisons test was used in (**a**-**c**). Body weight at each time point was analyzed using one-way ANOVA followed by Tukey’s multiple-comparison test between LLHA (0.25% Lys/1.00% Arg) and NLHA (0.00% Lys/1.00% Arg) (**b**). n.s., statistically nonsignificant, **p* < 0.05, ***p* < 0.01, and ****p* < 0.001.

**Fig. S3** Balancing Lys and Arg optimize survival in tumor-bearing mice, related to Figure 2.

(**a**-**c**) Tumor volume curves across nine dietary supplement groups with graded Lys-Arg combinations (n = 9-11). (**a**): 0.00% Lys/0.50% Arg, 0.25% Lys/0.50% Arg, 0.50% Lys/0.50% Arg. (**b**): 0.00% Lys/1.00% Arg, 0.25% Lys/1.00% Arg, 0.50% Lys/1.00% Arg. (**c**): 0.00% Lys/2.00% Arg, 0.25% Lys/2.00% Arg, 0.50% Lys/2.00% Arg. Tumor dimensions were measured daily after tumors became measurable, and tumor volume was calculated as 1/2 × L × W². Tumor volume was below 2000 mm³ on Day 21 and first exceeded 2000 mm³ on Day 22. Therefore, the Day 22 measurement was included as the endpoint value, and the animal was considered to have reached the humane endpoint at that time in (**b**).

(**d**) 3D surface response analysis of tumor volume from day 3 to 22 based on the data shown in (**a**-**c**). The x- and y-axes indicate Lys and Arg concentrations, respectively, and the z-axis represents tumor volume.

Data were reported as mean ± SEM. Two-way ANOVA with Sidak multiple comparisons test was used in (**a**-**c**). n.s., statistically nonsignificant, **p* < 0.05, ***p* < 0.01, and ****p* < 0.001.

**Fig. S4** Balancing Lys and Arg optimize survival in tumor-bearing mice, related to Figure 2.

(**a**-**d**) Survival curves across nine dietary supplement groups with graded Lys-Arg combinations (n = 7-11). (A): 0.00% Lys/0.50% Arg, 0.25% Lys/0.50% Arg, 0.50% Lys/0.50% Arg. (**b**): 0.00% Lys/1.00% Arg, 0.25% Lys/1.00% Arg, 0.50% Lys/1.00% Arg. (**c**): 0.00% Lys/2.00% Arg, 0.25% Lys/2.00% Arg, 0.50% Lys/2.00% Arg. (**d**) 0.50% Lys/0.50% Arg, 0.50% Lys/1.00% Arg, 0.50% Lys/2.00% Arg. Survival was monitored after tumor inoculation until the ethical endpoint or the end of observation. The difference in sample size between body weight/tumor volume analyses and survival analysis was due to spleen collection for scRNA-seq. On day 19, three mice from the LLHA group and three from NLHA were sacrificed for spleen collection and were therefore excluded from the survival analysis, while their body weight and tumor volume data before day 19 were included in the corresponding analyses.

Data were reported as mean ± SEM. A log-rank (Mantel-Cox) test was used in (**a**-**d**). n.s., statistically nonsignificant, **p* < 0.05.

**Fig. S5** qPCR validation of selected transcriptional signatures in LLHA and NLHA groups, related to Figures 4 and 6.

(**a**) Relative expression of *Rps6*, a downstream component related to mTORC1 activity and translational regulation, validating the mTOR-related signature observed in plasma cells.

(**b**-**c**) Relative expression of hemoglobin-related genes *Hba-a1* (**b**) and *Hbb-bt* (**c**), validating the hemoglobin-related gene-expression pattern observed in M1 macrophages in Figure 6g.

(**d**) Relative expression of *Rps29*, validating the ribosomal/translational signature observed in M1 macrophages in Figure 6j.

Data are presented as mean ± SEM. Unpaired two-tailed t-test was used for statistical analysis. **p* < 0.05. NLHA, 0% Lys/1% Arg; LLHA, 0.25% Lys/1% Arg.

**Fig. S6** qPCR validation of selected transcriptional signatures in LLHA and NLHA groups.

Relative expression of *Slc7a1* in spleen tissues from NLHA- and LLHA-treated mice.

Data are presented as mean ± SEM. Unpaired two-tailed t-test was used for statistical analysis. NLHA, 0% Lys/1% Arg; LLHA, 0.25% Lys/1% Arg. n.s., no significance.
